# The EPA-council of national associations in implementing digital mental health across Europe: Opportunities and challenges

**DOI:** 10.1192/j.eurpsy.2021.59

**Published:** 2021-08-13

**Authors:** S. Vahip

**Affiliations:** Department Of Psychiatry, Ege University Medicine Faculty, Izmir, Turkey

## Abstract

**Abstract Body:**

Digital solutions and interventions for mental health have increasingly been taking place in many societies in the last several decades. There are significant differences among countries due to economical and organizational situations. On the other hand, despite digital gap, there is a significant increase in the use of telepsychiatry and e-mental health applications with the Covid-19 pandemic throughout the world. Experiences of this pandemic times make many opportunities and challenges more apparent in this field. Safety and security, legislation, regulations, good practice standards, evidence based data, ethics and education are several of main areas of needs. EPA with the Council of National Psychiatric Associations (NPAs) is one of the crucial organizations in Europe which may play an important role to work on these challenges and opportunities. EPA-Council of NPAs consists of 44 associations represent psychiatrists (and other mental health workers in some) from 40 European countries. NPAs are crucial organisations in contact with local and national mental health stakeholders; competent in national, local, authentic and cultural issues and sensitivities; and could serve as crucial junctions for Europewide policies and their widespread implementations. Some reflections on challenges and opportunities from the Council of NPAs will be presented, based on a rapid survey and personal communications with presidents and official representatives of NPAs for future perspectives.

**Disclosure:**

No significant relationships.

